# A Serine-Threonine Kinase (StkP) Regulates Expression of the Pneumococcal Pilus and Modulates Bacterial Adherence to Human Epithelial and Endothelial Cells *In Vitro*

**DOI:** 10.1371/journal.pone.0127212

**Published:** 2015-06-19

**Authors:** Jenny A. Herbert, Andrea M. Mitchell, Timothy J. Mitchell

**Affiliations:** Institute of Microbiology and Infection, School of Immunity and Infection, University of Birmingham, Birmingham, England, United Kingdom; Instituto Butantan, BRAZIL

## Abstract

The pneumococcal serine threonine protein kinase (StkP) acts as a global regulator in the pneumococcus. Bacterial mutants deficient in StkP are less virulent in animal models of infection. The gene for this regulator is located adjacent to the gene for its cognate phosphatase in the pneumococcal genome. The phosphatase dephosphorylates proteins phosphorylated by StkP and has been shown to regulate a number of key pneumococcal virulence factors and to modulate adherence to eukaryotic cells. The role of StkP in adherence of pneumococci to human cells has not previously been reported. In this study we show StkP represses the pneumococcal pilus, a virulence factor known to be important for bacterial adhesion. In a serotype 4 strain regulation of the pilus by StkP modulates adherence to human brain microvascular endothelial cells (HBMEC) and human lung epithelial cells. This suggests that the pneumococcal pilus may play a role in adherence during infections such as meningitis and pneumonia. We show that regulation of the pilus occurs at the population level as StkP alters the number of pili-positive cells within a single culture. As far as we are aware this is the first gene identified outside of the pilus islet that regulates the biphasic expression of the pilus. These findings suggest StkPs role in cell division may be linked to regulation of expression of a cell surface adhesin.

## Introduction

*Streptococcus pneumoniae* is normally found as a commensal in the nasopharynx of humans, colonising 10% to 40% of adults and children respectively [1]. This bacterium can also cause a number of invasive diseases including meningitis, septicaemia, pneumonia and otitis media. The organism is the worlds biggest killer of children under the age of five, and is a huge burden on the health services worldwide [2]. Current vaccines target the pneumococcal capsule. However there are over 90 different capsule types and only a small proportion of these can be included in the vaccine composition [3]. Vaccine replacement and capsule switching adds to the limitations of this type of vaccine in the longer term [4–8]. The high cost of this vaccine also prevents its usage in countries with the highest burden of disease.

The ability of the pneumococcus to cause invasive disease is attributed to its large cache of virulence factors (see review [9]). This includes genes encoding two types of pilus (type 1 and type 2). The type 1 pneumococcal pilus is encoded on a 12kb genomic islet (PI-1) and is present in approximately 30% of pneumococcal strains [10]. The type 2 pilus is encoded on a 6.6kb island (PI-2) encoding 5 genes and is present in approximately 16% of strains [11]. Pilus 2 expression has a marginal effect on bacterial adhesion to epithelial cells [11]. The PI-1 islet contains 7 genes, three encoding pilus structural proteins (RrgA, RrgB and RrgC), three encoding the sortase enzymes that are required for pilus assembly (SrtB, SrtC and SrtD) and one encoding a positive regulator of the islet (RlrA) [12,13]. There are 4 promoters; one upstream of *rlrA*, *rrgA*, *rrgB* and *srtB* which may allow differential expression of these genes [14]. The type 1 pilus has been shown to play a role in host cell adhesion, biofilm formation, and pathogenesis of pneumonia and is being studied as a potential vaccine candidate [12,15–18]. Recent findings have also implicated the type 1 pilus in modulating host immune defence by interacting with TLR2 and complement receptor 3 [19,20]. Regulation of the type 1 pneumococcal pilus is complex potentially involving two transcriptional regulators and six two-component signal transduction systems (TCS) [21–25]. The pilus also shows a biphasic pattern of expression with roughly 30% of bacteria in a single population expressing the pilus on the cell surface at any one time. The proportion of cells expressing the pilus is strain dependant. The regulatory mechanism controlling this biphasic pilus expression is complex and is mediated by expression of *rlrA* and a positive feedback loop of the regulator RlrA [26,27].

The pneumococcus has a single eukaryotic like serine/threonine protein kinase which is a global regulator that regulates genes involved in natural competence development, cell wall biosynthesis, oxidative stress response and metabolism [28,29]. The importance of this protein has been shown by gene deletion experiments. A *stkP* deletion mutant has decreased ability to survive under stress induced conditions (heat/ osmotic/ oxidative and acid stress), reduced transformation efficiency and decreased virulence *in vivo* in a bacteraemia and pneumonia model of infection [29,30].

StkP contains an N-terminal kinase domain joined by a hydrophobic linker to an extracellular domain [28,31] that consists of four penicillin binding protein and serine threonine kinase associated (PASTA) domains [32]. PASTA domains in penicillin binding protein 2x (PBP2x) are thought to recognise the amount of unlinked peptidoglycan and regulate the amount of cross-linking via its transpeptidase domain [33,34]. These domains are the target of β-lactam antibiotics blocking cell wall biosynthesis leading to bacterial cell death [35,36]. The PASTA domains in StkP have also been shown to bind to synthetic peptidoglycan and β-lactam antibiotics [32,37]. This binding has been shown to directly affect the activity of the kinase domain, and the level of phosphorylation of target proteins. Furthermore, the PASTA domains have been shown to be important in localisation of StkP to the cell septum [37,38].

StkP modulates expression via phosphorylation of target proteins on a serine or threonine residue [39]. Current known targets of StkP include a number of cell division proteins and it has also been shown to phosphorylate response regulator CbpS (RR06) and response regulator RitR (ORR) [40–42].

Here we show that StkP represses expression of the type 1 pneumococcal pilus in a serotype 4 strain. This regulation modulates adherence to human brain microvascular endothelial cells and human lung epithelial cells. Complementation of an *stkP* knockout mutant with the full length *stkP* gene reverted the level of adherence to wild-type. Expression of a variant of the StkP protein lacking a single PASTA domain caused partial reversion of adherence demonstrating the importance of the PASTA domain to StkP signalling. This regulation of the pilus was shown to occur at the population level.

## Materials and Methods

### Bacterial strains and growth conditions

The bacterial strains used in this study are described in S1 Table. S.*pneumoniae* strains were cultured on blood agar base (BAB, Oxoid) with 5% Horse blood (E&O laboratories) at 37°C with 5% CO_2_. Liquid cultures were prepared by growth at 37°C in brain heart infusion broth (Oxoid) to OD_600nm_ 0.6. Media were supplemented with kanamycin (400μg/ml), spectinomycin (200μg/ml) or chloramphenicol (10μg/ml) depending on the selectable marker present in the strain. *E*.*coli* strains were grown on Luria Bertani (LB) agar or in LB broth containing the appropriate antibiotic, ampicillin (100μg/ml) or kanamycin (100μg/ml).

### *In vitro mariner* mutagenesis

MarC9 transposase was purified as previously described [43,44]. The reaction mixture consisted of 1μg of *stkP* PCR product, 10μl 2x transposition buffer (10% glycerol, 2mM DTT, 25mM Hepes pH 7.9, 250μg/ml BSA, 100mM NaCl, 10mM MgCl_2_), 1μg pR412 plasmid DNA, 0.5μl transposase enzyme and made to 20μl with PCR grade water. The reaction mix was incubated for 6 hours at 30°C. The reaction was cleaned using the Wizard SV gel and PCR clean up system (Promega) as per manufacturers guide and heated for 10 minutes at 75°C to inactivate the transposase. To the eluate 10μl 10x T4 DNA reaction buffer, 1.5μl 2mM dNTPS, 2μl T4 DNA polymerase (3U/μl) (NEB) and 0.5μl 10mg/ml BSA were added and incubated at 16°C for 30 minutes followed by 75°C for 10 minutes to inactivate the enzyme. Finally 2μl of 1mM NAD+ (β-Nicotinamide adenine dinucleotide, NEB) and 4μl *E*.*coli* DNA ligase (NEB) was added, incubated at 16°C overnight and then stored at 4°C until transformation. The whole reaction mix was subsequently used to transform an unencapsulated TIGR4 strain.

### Construction and verification of gene knockouts and complemented strains

All primers used for construction of mutants and complemented strains are listed in S2 Table. Construction of T4Δ*stkP* was performed via *in vitro mariner* mutagenesis. The full *stkP* gene was amplified from TIGR4 using primers stkPF FL and stkPR. *In vitro mariner* mutagenesis reactions were performed as described above. The initial mutant was constructed in an unencapsulated TIGR4 strain due to its increased transformation efficiency [45]. The resulting mutant contained a transposon insertion in *stkP* containing a spectinomycin resistance cassette. The position and directionality of the insert was confirmed by using primers MP127 and MP128. To transfer the insertion into TIGR4 primers stkPF FL and stkPR were used to amplify stkP containing the transposon insertion, which was subsequently transformed into TIGR4 creating T4Δ*stkP*.

### Splice overlap PCR

Construction of an RrgB deletion mutant was performed via splice overlap PCR replacing the whole *rrgB* gene with a kanamycin resistance cassette amplified from pR410 plasmid [46]. Three primer pairs were required amplifying the upstream (0462F-25C/ 0462 R) and downstream (0464F/ 0464 R-34R) of *rrgB* and the kanamycin resistance cassette from pR410 (KANGB-F/ KANGB-R). Fragments were transformed into TIGR4 and selected on BAB containing kanamycin. Gene deletion was confirmed by sequencing. For construction of T4Δ*stkP*Δ*rrgB* the Δ*rrgB* fragment was amplified from T4Δ*rrgB* and transformed into T4Δ*stkP*.

### Construction of StkP complemented strains

StkP complemented strains were constructed placing either the full length StkP amplified from TIGR4 or 3^rd^ PASTA domain deletion StkP (amplified from strain Xen35) downstream of a strong promoter chromosomally inserted into SP_1886 in T4Δ*stkP*. A modified version of pCEP2 [47] was used in which a strong promoter identified from RNA-seq analysis of TIGR4 was inserted. To create T4Δ*stkP*∇ST and T4Δ*stkP*∇XST plasmids pCP2 ST and pCP2 XST were transformed into T4Δ*stkP* respectively. Integration into the genome at the position of SP_1886 was confirmed by PCR using primers flanking the insertion site (S2 Table).

### Pneumococcal transformation

Pneumococcal transformation was performed as described in [48], except CSP-2 was used and BAB plates supplemented with the desired antibiotic, for T4Δ*stkP* spectinomycin (200μg/ml), T4Δ*rrgB* kanamycin (400μg/ml) and T4Δ*stkP*∇ST/ T4Δ*stkP*∇XST chloramphenicol (10μg/ml). For plasmid and PCR product transformation 1μg of DNA was used. For *in vitro* mariner mutagenesis the whole final reaction was used for transformation.

### Isolation of pneumococcal RNA

For RNA extraction pneumococcal cultures were grown in triplicate in BHI to OD_600nm_ 0.6. RNA extraction was performed as described in [22], with the following exceptions. After the initial lysis steps extraction was performed using the RNeasy mini kit (Qiagen), as per the manufacturers instructions. Post extraction a further DNase step was performed using the TURBO DNA-free kit (Ambion, Life Technologies, UK) as per the manufacturers guide. RNA was stored at -80°C until required.

For cDNA synthesis 2μg of total RNA was used and 1μl of random primers added (Life Technologies) and made to 11μl with nuclease free water. Samples were incubated at 70°C for 10 minutes and then snap cooled on ice. To each sample 5μl 5x first strand buffer, 2.5μl DTT (100mM), 2.3μl dNTP mix (5mM dGTP/ dATP/ dTTP/ dCTP); 1.7μl nuclease free water and 2.5μl Superscript 11 (200U/μl) were added. Samples were incubated at 25°C for 10 minutes followed by 45°C for 90 minutes.

### RT-PCR

All primers used for RT-PCR analysis are described in S3 Table. cDNA synthesis was performed as described above.

Real-time PCR was performed using FastStart Universal SYBR green master mix (ROX) (Roche) as per manusfacturers instructions. All samples were analysed on a Chromo4 system CFB-3240 (Bio-Rad, USA). Reaction conditions consisted of an initial incubation of 2 minutes at 50°C, followed by 10 minutes at 95°C. 40 cycles of 95°C for 15 seconds, 55°C for 30 seconds and 72°C for 30 seconds were performed followed by a final 5 minutes at 72°C. *gyrA* was used as an internal control to normalise for cDNA synthesis variations.

Analysis was performed in Opticom Monitor version 3.1. Background was subtracted in the software from No-RT controls and replicates grouped together with at least two replicates used for analysis. Data was analysed using the 2^-ΔΔC^_T_ method [49]. Graphical data representation was performed in Prism version 4.0b (GraphPad Software), each bar representing the sample replica means ± standard deviation error bars.

### Western blotting

For all western blots *S*.*pneumoniae* strains were grown to OD_600nm_ 0.6 in BHI. The bacterial extracts were run on NuPAGE® Novex 4–12% Bis-Tris gels and proteins were transferred using the iBlot® module. The membrane was blocked and then incubated with a 1/4000 dilution of the primary antibody. Primary antibody either consisted of an in house Mouse Anti- RrgB, Rabbit Anti- RrgA, Rabbit Anti- StkP, Rabbit Anti- PhpP and Rabbit Anti- GroEL (*E*.*coli*) pAb (Enzo Life Sciences, UK). Primary antibody was detected using a 1/20,000 dilution of the HRP labelled secondary antibody Goat Anti-Rabbit IgG HRP linked F(ab)_2_ (GE Healthcare) or Goat Anti-Mouse IgG HRP (Southern Biotech) and developed using Immobilon Western Chemiluminescent HRP substrate (Millipore).

### Flow cytometry

*S*.*pneumoniae* strains were fixed in 1ml 2% paraformaldehyde (Sigma-Aldrich, UK). Antibody staining was performed as described in [27]. Double antibody staining was performed using Mouse Anti- RrgB polyclonal (raised in house) and Rabbit Anti- Type 4 capsule (Statens Serum Institute) followed by secondary antibody staining with Goat Anti-Rabbit IgG (H&L chain specific) Allophycocyanin (APC) conjugate (Southern Biotech) or Goat Anti-Mouse IgG (γ chain specific) Fluorescein (FITC) Conjugate (Southern Biotech).

Labelled samples were analysed on a FACScalibur flow cytometer using CellQuest-Pro software (BD biosciences). FACS analysis was performed in FlowJo 9.4.10 for Macintosh (Tree Star).

### Adherence assay

HBMEC (Human brain microvascular endothelia cells) were grown in Advanced RPMI 1640 media (Life technologies) supplemented with 20% FBS (Fetal bovine serum (EU approved), Biosera), 2mM L-Glutamine (Sigma Aldrich), 1% 100X Penicillin streptomycin solution (Sigma Aldrich,), and 1% Fungizone Antimycotic (Gibco). A549 (Human lung epithelial carcinoma cell line, ATCC-CCL-185) cells were grown in Hams F12K (Kaighns modification) media (Life Technologies) supplemented with 10% FBS, Penicillin streptomycin and Fungizone as above.

For adherence assay all cell lines were grown in the same media as stated above however the penicillin streptomycin solution and Fungizone® was omitted. All adherence assays were performed in 24 well microtitre plates. 2x10^5^ viable cells were seeded into each well and made to 1ml with media. The plates were incubated at 37°C in 5% CO_2_ for 48 hours (cells confluent). 10^7^ CFU of *S*.*pneumoniae* taken directly from a frozen bacterial stock were seeded in 1ml tissue culture media into a single well containing tissue culture cells. The assay was incubated at 37°C in 5% CO_2_ for 2 hours when viable counts were performed giving the number of non-adherent bacteria. Cells were then washed 3 times and solublised in 0.0125% Triton-X-100 (Sigma-Aldrich) and the percentage of adherent bacteria calculated. Graphical presentation and statistical analysis was performed in Prism version 4.0b (GraphPad Software). Statistical analysis was performed using a one-way ANOVA with Tukey’s multiple comparison test.

## Results

A gene deletion of *stkP* (T4Δ*stkP*) was constructed in a serotype 4 strain (TIGR4). Complemented strains were constructed expressing full length StkP (TIGR4) or the 3^rd^ PASTA domain deleted allelic variant of StkP (referred to as XST) [47,50,51]. The full length and allelic variant amino acid sequence of StkP are shown in S1 Fig. Deletion of the whole 3^rd^ PASTA domain in this strain has occurred between two almost identical 10 amino acid repeats located at the end of the second and third PASTA domain. The kinase is highly conserved within the pneumococcus and shows 99.5% amino acid sequence homology between strains [52]. Use of this natural variant avoided the need for complex cloning. There is currently no crystal structure available to evaluate what the effect of this deletion may have on StkP structure. However schematic models imply the PASTA domains likely span the width of the peptidoglycan cell wall [38]. Removal of one of these domains would likely shorten the length the domains span beyond the cell wall and may affect interaction between the PASTA domains and their extracellular stimulus, in turn altering kinase activation.

### Regulation of the pilus by StkP

**Fig 1:**
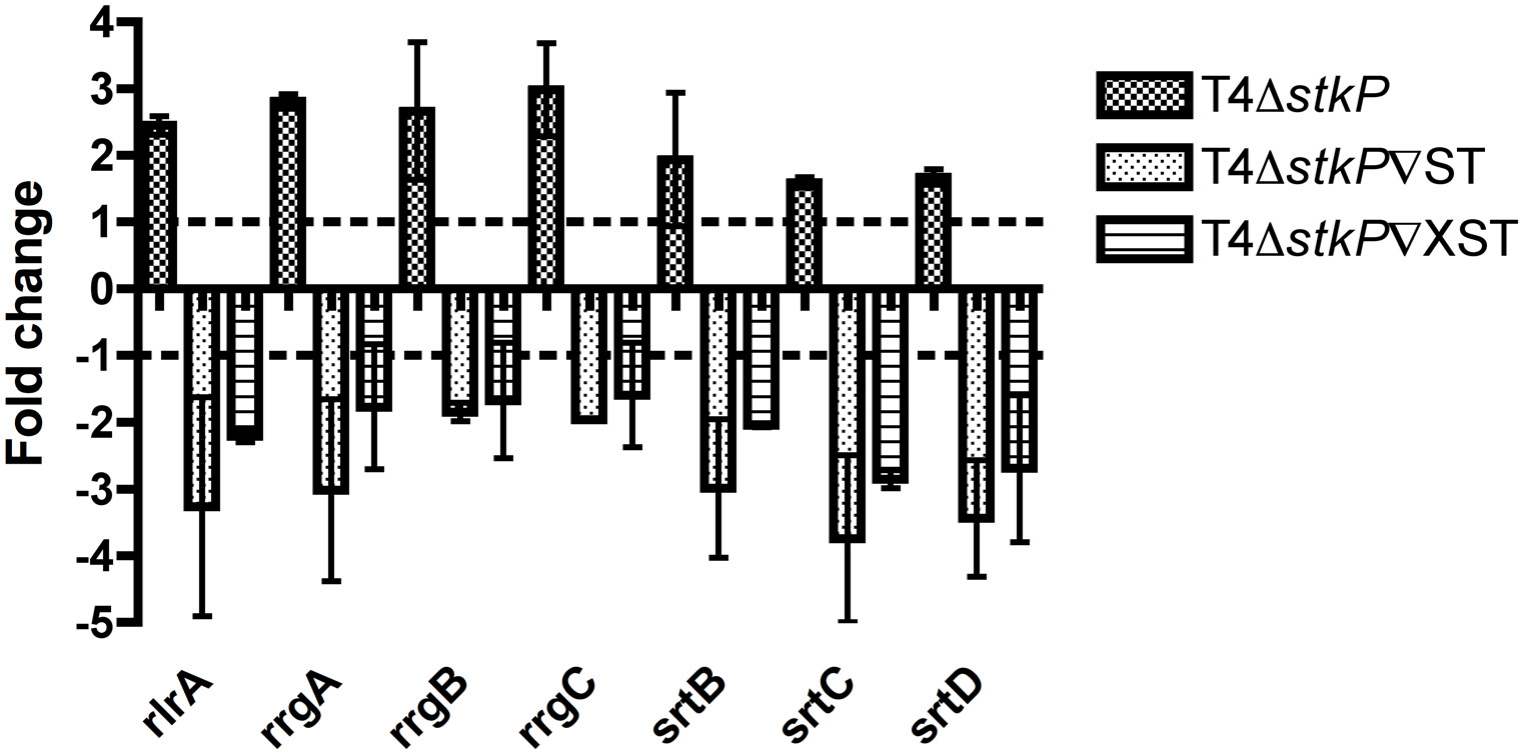
Pilus gene expression in T4Δ*stkP* and StkP complemented strains. Graph shows RT-PCR expression of the genes present on the whole pilus islet (*rlrA*, *rrgA*, *rrgB*, *rrgC*, *srtB*, *srtC*, *srtD*) in T4Δ*stkP*, T4Δ*stkP*∇ST and T4Δ*stkP*∇XST compared to TIGR4. Fold change represents that of the mutant strain compared to TIGR4. Each bar represents the average of three replicas and errors bars the standard deviation.

Initial experiments were performed evaluating expression changes in the 7 pilus islet genes in the single StkP knockout in TIGR4 and the StkP complemented strains (Fig 1) [13,17].

In the StkP knockout RT-PCR analysis showed an up-regulation of all genes present on the pilus islet compared to TIGR4, suggesting that StkP normally functions to repress expression of the pilus islet genes. When the full length StkP was expressed in T4Δ*stkP* (T4Δ*stkP*∇ST) this reverted the effects and there was a decrease in the expression of the pilus islet genes (Fig 1). When the allelic variant was expressed in T4Δ*stkP* (T4Δ*stkP*∇XST) there was also a drop in expression of the pilus islet genes but not to the same level as that seen in T4Δ*stkP*∇ST.

**Fig 2:**
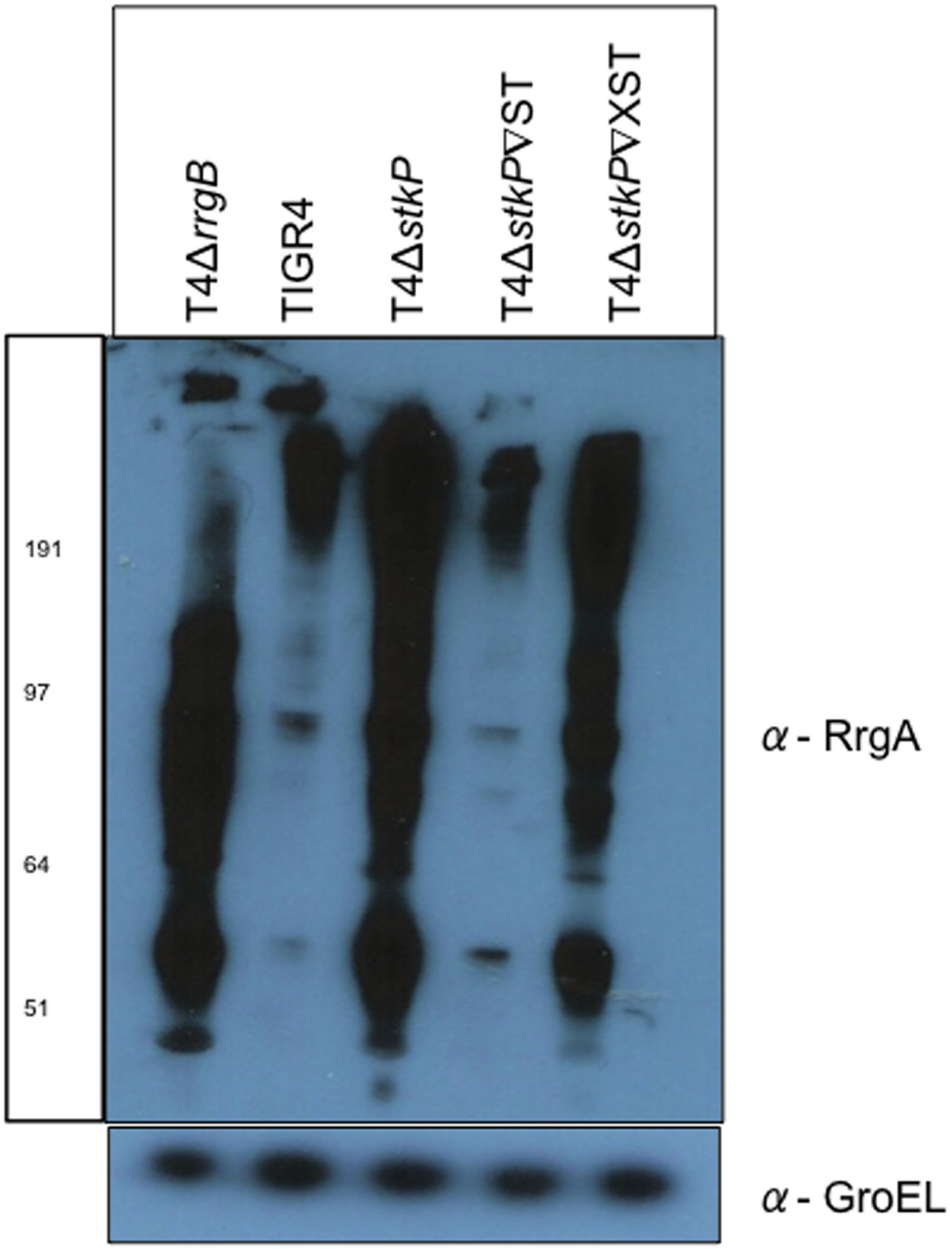
RrgA protein expression in T4Δ*stkP* and StkP complemented strains. Western blotting analysis was performed on TIGR4, T4Δ*stkP*, T4Δ*stkP*∇ST, T4Δ*stkP*∇XST and T4Δ*rrgB* at OD 0.6 (OD_600nm_) looking at expression of RrgA in all strains (α-RrgA antibody). Equal protein loading was confirmed by equal expression of GroEL (α-GroEL antibody).

**Fig 3:**
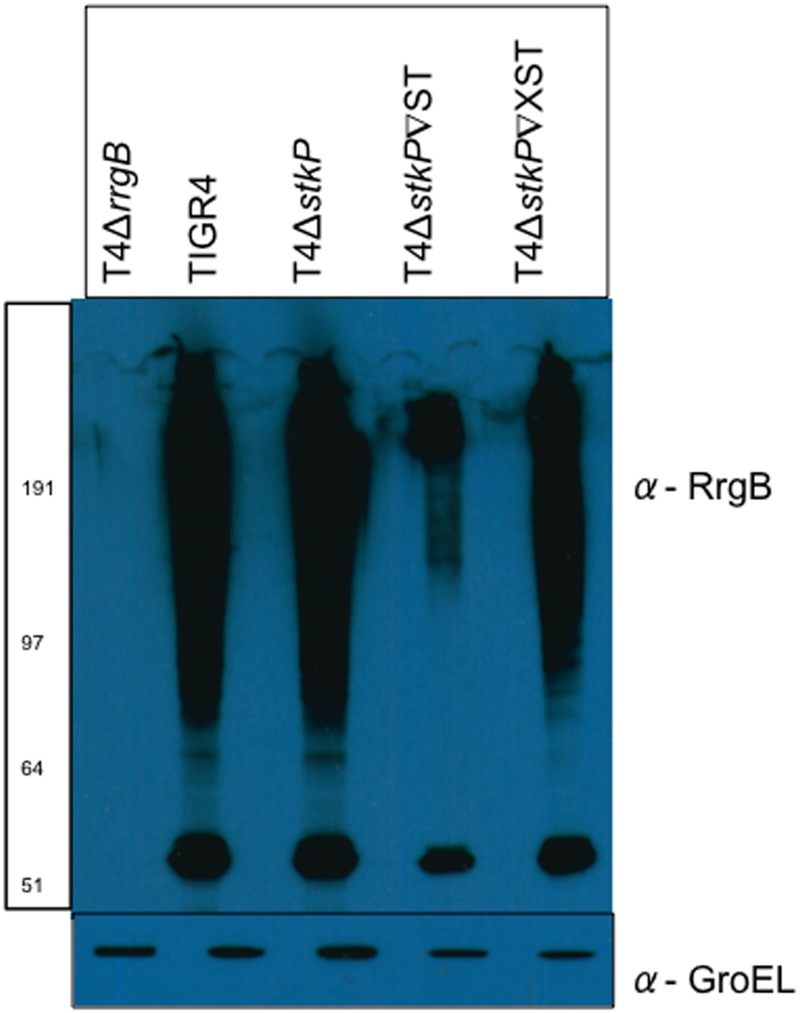
RrgB protein expression in T4Δ*stkP* and StkP complemented strains. Western blotting analysis was performed on TIGR4, T4Δ*stkP*, T4Δ*stkP*∇ST, T4Δ*stkP*∇XST and T4Δ*rrgB* at OD 0.6 (OD_600nm_) looking at expression of RrgB in all strains (α-RrgB antibody). Equal protein loading was confirmed by equal expression of GroEL (α-GroEL antibody).

Western blot analysis was also performed to evaluate if the change in gene expression observed correlated to changes in total protein level. Changes in protein levels were evaluated in T4Δ*stkP*, T4Δ*stkP*∇ST, T4Δ*stkP*∇XST, T4Δ*rrgB* and TIGR4 for the pilus adhesin RrgA (Fig 2) and the major pilin RrgB (Fig 3), which are key for adhesion to host cells and to pilus assembly respectively [12,53]. Blots for both RrgA and RrgB show smears due to the majority of both proteins being found as polymers. The single low molecular weight band shows the monomeric form of the proteins. A large increase in RrgA protein levels in T4Δ*stkP* compared to TIGR4 was observed. When StkP (T4Δ*stkP*∇ST) was complemented there was a large decrease in RrgA protein levels back to levels similar to those in TIGR4. This was not observed when the allelic variant of StkP was expressed in T4Δ*stkP* (T4Δ*stkP*∇XST) and only a small drop in RrgA protein levels was observed compared to T4Δ*stkP*.

RrgB protein levels were not clearly different in the kinase knockout compared to TIGR4. However when complementing this mutant (T4Δ*stkP*∇ST) there was a clear drop in RrgB protein levels compared to TIGR4 and T4Δ*stkP* suggesting that overexpression of the kinase suppresses RrgB expression. When complementing this mutant with the allelic variant of StkP (T4Δ*stkP*∇XST) the drop in RrgB levels was not as prominent but there was still a decrease compared to TIGR4 and T4Δ*stkP*.

We observed several discrepancies between gene expression data and levels of protein observed by western blot. This is presumably related to the different rates of accumulation of pilins. Very little is known about regulation of stability and degradation of these proteins. However there is some indication that regulation also occurs at the protein level with RlrA and RrgA interacting to modulate expression levels of the islet [27].

### Co-regulation of the kinase and phosphatase

**Fig 4:**
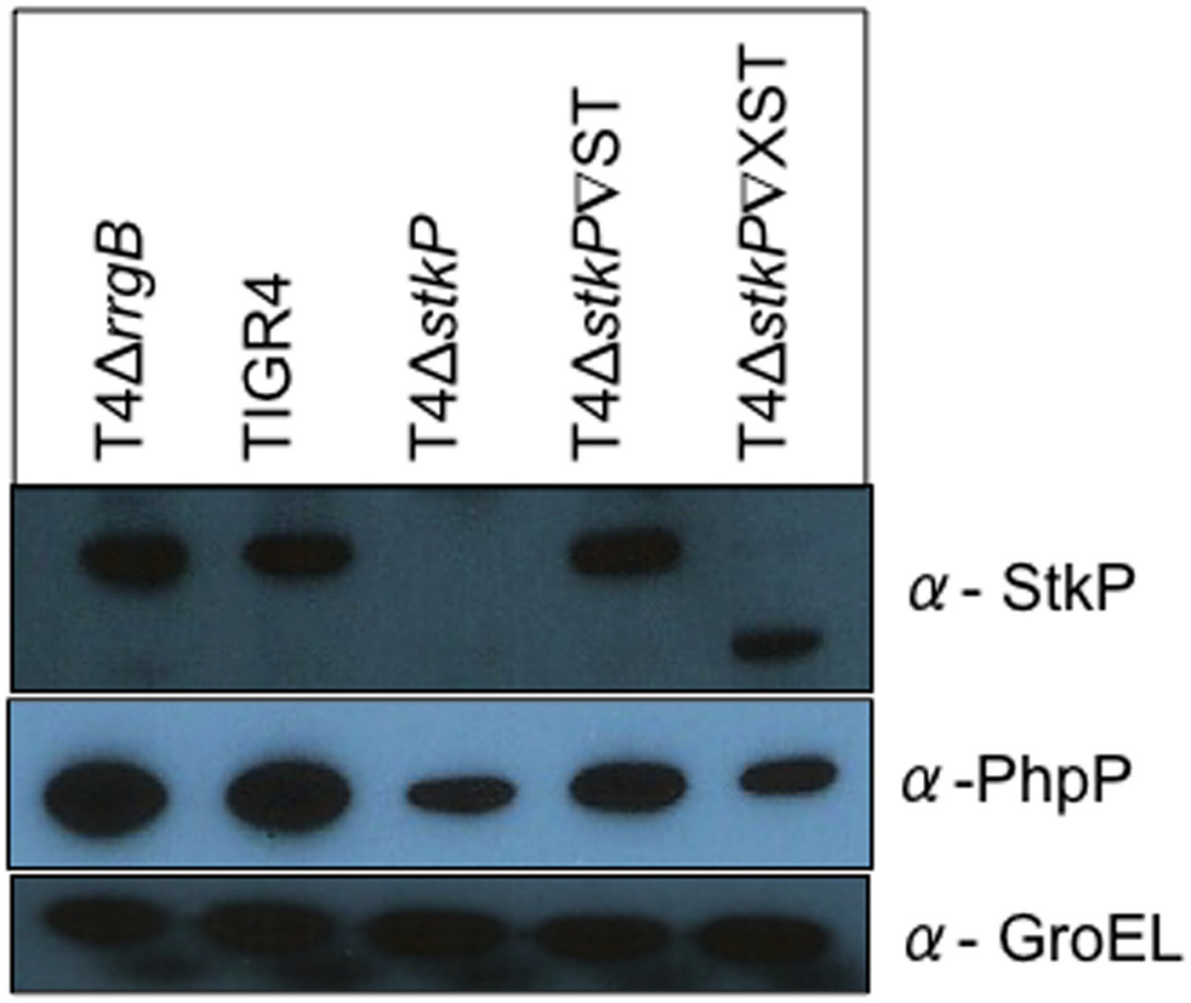
Stkp and PhpP protein expression in T4Δ*stkP* and StkP complemented strains. Western blotting analysis was performed on TIGR4, T4Δ*stkP*, T4Δ*stkP*∇ST, T4Δ*stkP*∇XST and T4Δ*rrgB* at OD 0.6 (OD_600nm_) looking at expression of StkP and PhpP in all strains (α-StkP/ -PhpP antibody). Equal protein loading was confirmed by equal expression of GroEL (α-GroEL antibody).

Western blot analysis was performed on strains to evaluate the protein levels of the kinase and phosphatase (Fig 4). As expected no StkP was observed in T4Δ*stkP*. When T4Δ*stkP* was complemented with wild type StkP the levels of StkP observed was similar to that of TIGR4 (Fig 4). When the allelic variant was expressed in T4Δ*stkP* a smaller StkP band was observed due to the deletion of the 3^rd^ PASTA domain. This band was also less dense suggesting less StkP is present, however this is possibly due to the reduced binding of the StkP antibody as the PASTA domains are the immunogenic region of StkP [52,54]. This is further validated via RT-PCR analysis of the expression levels of the kinase in T4Δ*stkP*∇ST and T4Δ*stkP*∇XST which shows similar levels of expression. Levels of the phosphatase are also similar in T4Δ*stkP*, T4Δ*stkP*∇ST, T4Δ*stkP*∇XST (S2 Fig).

*stkP* and *phpP* are found adjacent on the chromosome and their functions have been closely linked [28,39,55]. Further StkP is required for localisation of PhpP to the midcell so deletion of StkP will in turn affect PhpP activity [37]. Therefore it is not surprising that there is altered protein level of the phosphatase in the kinase mutants. With all three mutants showing lower amounts of PhpP than that seen in TIGR4. The levels are higher when the wild type kinase is expressed in T4Δ*stkP* (T4Δ*stkP*∇ST) at an unlinked part of the chromosome [47]. This would suggest this is not a direct polar effect of deletion of *stkP* in TIGR4. Rather it is a consequence of altered StkP levels, which are closely regulated to levels of PhpP [39].

### Modulation of adherence by StkP in TIGR4

**Fig 5:**
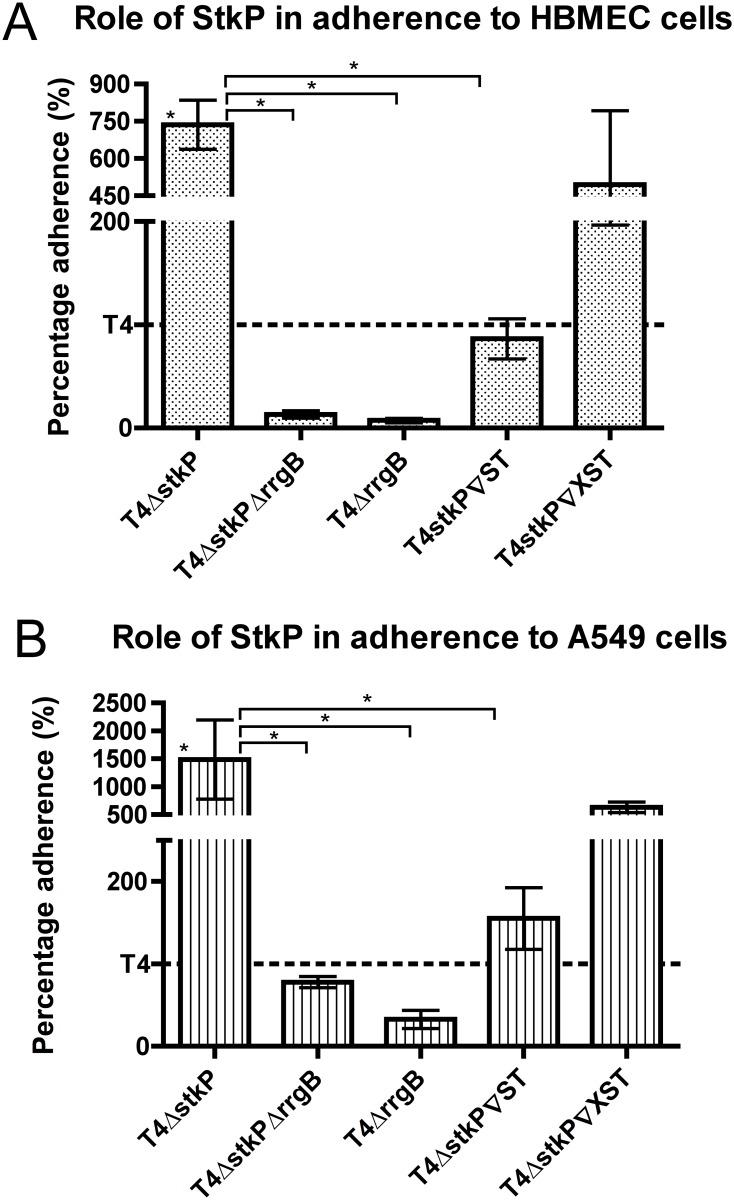
Adherence of T4Δ*stkP* and StkP complemented strains to different cell lines. Adherence of strains TIGR4, T4Δ*stkP*, T4Δ*stkP*Δ*rrgB*, T4Δ*stkP*∇ST, T4Δ*stkP*∇XST and *T4*Δ*rrgB* was assessed to HBMEC (A) and A549 (B) cell lines. Data is represented as percentage adherence relative to that of TIGR4 (100%, dashed line), each bar is an average of three replicas and the error bars represent the standard error of the mean. Statistical analysis was performed using a 1-way ANOVA with a Tukeys testing correction, * P<0.05. * above the bar represent statistical significance compared to TIGR4 (not represented as a bar on the graphs).

To evaluate the effect of altered pilus levels on adherence in the StkP mutant and complemented strains, their ability to adhere to Human Brain Microvascular Endothelial cells (HBMEC) and A549 (Human lung epithelial carcinoma cells) were assessed (Fig 5). These cell lines can be used to model *in vitro* adherence of pneumococci to host cells during meningitis and pneumonia respectively.

Deletion of the kinase gene caused an increase in adherence to both cell lines. Deletion of RrgB in T4Δ*stkP* (T4Δ*stkP*Δ*rrgB*) abolished this increase in adherence showing the effect is solely due to an increase in pilus expression and not other adhesins. T4Δ*stkP*Δ*rrgB* adherence capabilities were not different to that of T4Δ*rrgB* and TIGR4.

When the wild type kinase was expressed in T4Δ*stkP* the adherence to both cell lines reverted to the levels seen with TIGR4. When the allelic variant of StkP was expressed in T4Δ*stkP* there was a trend towards a drop in adherence to both cells lines but this was not significant. Similar adherence patterns of T4Δ*stkP* were observed also to Detroit 562 cells (nasopharyngeal carcinoma cell line) (S3 Fig.).

### Regulation of the pilus by StkP at the population level

StkP modulates pilus levels through expression changes, which in turn modulates adherence. It is not known if this is due to varying the number of pili on a single cell or the number of cells that have pili. Recent findings have shown that the number of pili-positive cells within a population can vary, although currently no genes have been shown to modulate this outside the pilus islet genes [26,27]. This biphasic pattern of expression of the pilus is mediated by expression of *rlrA* and the regulator protein RlrA is part of a positive feedback mechanism for pilus expression in individual bacterial cells [26,27].

**Fig 6:**
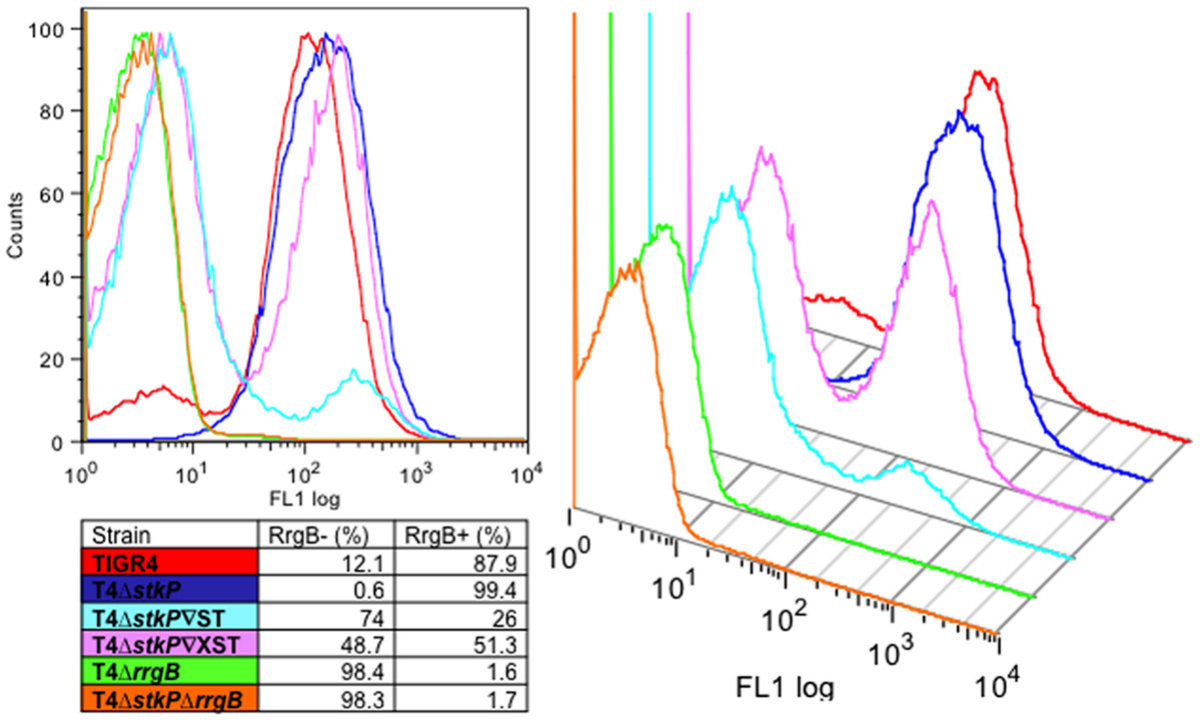
RrgB surface expression in T4Δ*stkP* and StkP complemented strains. Flow cytometry was performed on T4Δ*stkP*, TIGR4, T4Δ*stkP*∇ST, T4Δ*stkP*∇XST, T4Δ*stkP*Δ*rrgB* and T4Δ*rrgB*. All samples were initially gated on for being capsule positive (data not shown). This population was then gated on for RrgB positive. Histograms show negative (left) and positive (right) RrgB populations in each strain. Table shows the percentage RrgB positive and negative cells in a growing bacterial population.

Flow cytometry was used to evaluate the biphasic expression of pili. To deduce the number of cells in a growing population that had RrgB present on the cell surface the whole bacterial population was stained with anti-capsular antibody and the proportion of those expressing pilli were measured using an RrgB specific antibody (Fig 6).

TIGR4 bacteria were gated in the FACS using the anti-capsular antibody. Gating using the anti-RrgB fluorescence channel showed that 88% of the cells within this population were RrgB positive. In T4Δ*stkP* the proportion increased to 99% of cells being pili positive. When T4Δ*stkP* was complemented with StkP (T4Δ*stkP*∇ST) the proportion of pili-positive cells decreased, with 26% of bacteria in the capsule positive population also being RrgB positive. When the allelic variant of StkP (T4Δ*stkP*∇XST) was expressed 51% of cells were RrgB positive. These data suggest that StkP modulates the pilus at the population level.

## Discussion

In this study we show that StkP regulates expression of the pneumococcal pilus in TIGR4. We have shown that in strain TIGR4 the pilus modulates most of the adherence to human cells grown in culture and that the level of adhesion is mediated through the kinase StkP.

The pneumococcal pilus has previously been shown to promote adherence to A549 cells. Here we show it is also important for adherence to HBMEC cells suggesting a broad role of the pilus in adherence to different host cells [12,25,53]. Specifically we suggest from our findings that the pilus may aid adherence to brain endothelial cells during meningitis. This has been shown in *Streptococcus agalactiae* (GBS) where the pilus plays a role in adherence to HBMEC cells and has also been shown to aid penetration of the blood brain barrier [56,57]. However it is difficult to determine the effect of pilus expression in the *stkP* knockout on virulence as it causes other deleterious phenotypes, such as defects in cell division [29].

The role of the pilus in disease is still debated as presence of the pilus genes has not been linked to invasive disease or outbreak strains [58]. However the pilus locus has been shown to be more prevalent in the PCV7 serotypes and its re-emergence has been noted in Massachusetts in non-vaccine serotypes and is therefore thought to confer some advantage to these strains [58,59]. There is evidence to suggest that the pilus provides an advantage during colonisation due to its adhesive properties and its presence in strains has been shown to inversely correlate to carriage of *Staphylococcus aureus* [60]. Here we show that the pilus adhesin allows binding to endothelial and epithelial cells suggesting the role of the pilus is to aid colonisation. Once invasion of normally sterile sites occurs (meninges/ lungs) the pilus can also adhere to the cell types present within these niches. This is supported by the finding that the pilus adhesin RrgA shares homology to eukaryotic like integrin domains which are involved in cell attachment to extracellular matrix molecules (ECM) [61]. This suggests that these may be the target of RrgA.

Recent studies have shown that the pilus displays a biphasic pattern of expression. In TIGR4 30% of cells express the pilus on the cell surface however this varies between serotypes [26,27]. In our experiments using TIGR4, 88% of the bacterial population expressed the pilus. This difference may be due to differences in the TIGR4 strains used in different laboratories.

It has been shown that there are no gene expression differences between pili positive and negative bacteria other than the genes present on the pilus islet itself [26]. This biphasic pattern of expression has been observed in other Streptococci with *Streptococcus pyogenes* modulating pilus expression at the population level based on temperature [62]. This phenomenon in the pneumococcus is in part modulated by the binding of RrgA (adhesin) to RlrA (positive regulator of the islet), which prevents RlrA positively regulating expression of the pilus islet [27].

Little is currently known about pilus biogenesis and assembly kinetics, which may explain why in some instances we observe differences in gene expression and protein levels of the pilins in the kinase knockout and complemented strains.

A large increase in adherence of TIGR4 to both HBMEC (7.5 fold) and A549 (15 fold) was observed when StkP was deleted. Due to the fact there are only 11% more cells in the population that are pili positive this increase seems disproportionate. However the recent structure of the pilus shows that RrgA can also multimerise forming chains of the adhesin reaching beyond the RrgB backbone [61]. The fact we see a large increase in RrgA protein levels in the StkP mutants might suggest more branched pili are present which may increase the adhesive capabilities of the bacterial cells. This is supported by the fact the amount of RrgA protein in the different strains as determined by Western blotting directly correlated to the adherence capabilities of the strains.

The kinase is highly conserved within the pneumococcus and shows 99.5% amino acid sequence homology between strains [52]. Here we identified an allelic variant of the kinase, which is not present in any of the current genome sequenced strains [52]. Based on the fact the kinase acts as a repressor of the pilus, when expressing the allelic variant this repression was not as strong when the 3^rd^ PASTA domain is not present. Whether this deletion alters StkP function due to reduced activation of the kinase by a reduced ability to bind peptidoglycan precursors or by physically reducing the size of the extracellular domain that may reduce the proximity to its substrate would require further study.

The mechanism by which the kinase modulates pilus expression is still unclear but a hypothesis can be formed based on previous literature. The kinase has previously been shown to phosphorylate two pneumococcal response regulators, one being the orphan response regulator (SP_0376) and the other being the response regulator of TCS06 (SP_2193) [41,42]. RR06 has previously been shown to directly regulate the pneumococcal pilus through binding to the promoter region of *rlrA* [23]. Therefore it is possible that deletion of the kinase affects the phosphorylation state of RR06, which in turn alters pilus expression levels. In other bacteria there are numerous references to the interaction of serine threonine kinases phosphorylating RR of TCS pairs, which do so on a serine or threonine residue rather than the traditional aspartate residue, which the cognate HK phosphorylates [63–65].

In summary this study has shown that the single serine threonine protein kinase regulates the pneumococcal pilus in a serotype 4 and modulates adherence to HBMEC cells. This suggests that the pneumococcal type 1 pilus may play a role in meningitis as well as its already defined role in pneumonia and colonisation [12,23,53]. The study has also identified a new mode of regulation at the population level of the kinase and has identified the kinase as the first gene to modulate this type of expression for the pneumococcal pilus.

## Supporting Information

S1 Fig PASTA domains in StkP. Diagram shows the amino acid sequence of the extracellular PASTA domains present at the C-terminal end of StkP. A- shows the four extracellular PASTA domains present in TIGR4 StkP. In dark blue are the two 10 amino acid repeats present at the end of the 2^nd^ and 3^rd^ PASTA domain, where the recombination event occurred in a serotype 4 strain (Xen35) containing the *stkP* allelic variant. Amino acids in red represent the amino acids deleted in the StkP allelic variant. B- shows the StkP amino acid sequence in Xen 35, with the 3rd PASTA domain not present, and in dark blue the remaining 10 amino acid repeat.
(TIF)

S2 Fig *stkP* and *phpP* expression in T4Δ*stkP* and StkP complements. A- Graph shows RT-PCR expression of *phpP* in T4Δ*stkP*, T4Δ*stkP*∇ST and T4Δ*stkP*∇XST compared to TIGR4. Fold change represents that of the mutant strain compared to TIGR4. Each bar represents the average of three replicas and errors bars the standard deviation. B- Shows RT-PCR expression of *stkP* in T4Δ*stkP*∇XST compared to T4Δ*stkP*∇ST. Fold change represents that of T4Δ*stkP*∇XST compared to T4Δ*stkP*∇ST. Each bar represents the average of three replicas and errors bars the standard deviation.
(TIF)

S3 Fig Adherence of T4Δ*stkP* to Detroit 562 cells. Adherence of strains TIGR4, T4Δ*stkP*, T4Δ*stkP*Δ*rrgB* and *T4*Δ*rrgB* was assessed to Detroit 562 cell lines. Data is represented as percentage adherence relative to that of TIGR4 (100%, dashed line). Each bar is an average of at least two replicates and the error bars represent the standard error of the mean. Statistical analysis was performed using a 1-way ANOVA with a Tukeys testing correction, * P<0.01.
(TIF)

S1 Table Strains and plasmids used in this study (PDF)

S2 Table Primers and related information used for construction of knockout strains via *in vitro* mariner mutagenesis and splice overlap PCR and primers used to construct plasmids pCP2 ST and pCP2 XST. (PDF)

S3 Table Primers and related information used for RT-PCR analysis of pilus, *stkP* and *phpP* expression in T4Δ*stkP*, T4Δ*stkP*∇ST and T4Δ*stkP*∇XST. (PDF)
