# Supplementary figures and images for: A Serine-Threonine Kinase (StkP) Regulates Expression of the Pneumococcal Pilus and Modulates Bacterial Adherence to Human Epithelial and Endothelial Cells In Vitro
Source: PLoS One. 2015 Jun 19;10(6):e0127212. doi: 10.1371/journal.pone.0127212 (PMC4474723; doi:10.1371/journal.pone.0127212)

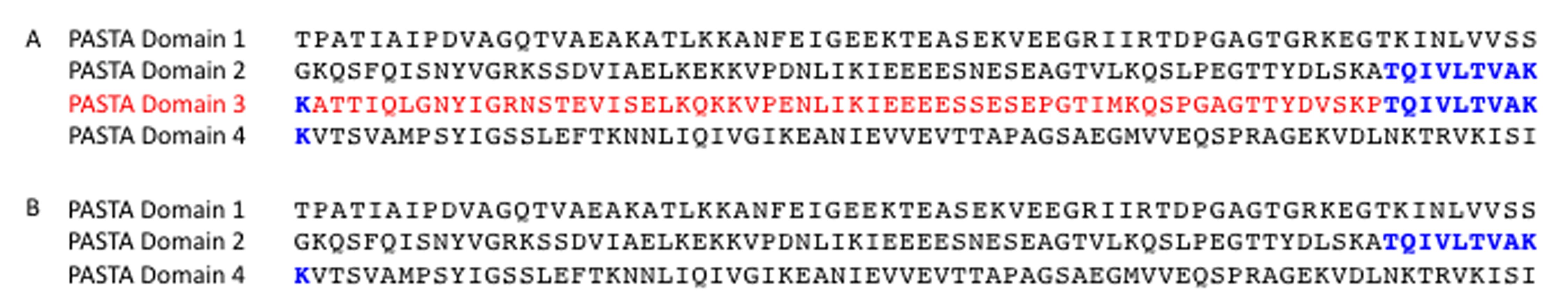

Supplement: S1 Fig — Diagram shows the amino acid sequence of the extracellular PASTA domains present at the C-terminal end of StkP. A- shows the four extracellular PASTA domains present in TIGR4 StkP. In dark blue are the two 10 amino acid repeats present at the end of the 2nd and 3rd PASTA domain, where the recombination event occurred in a serotype 4 strain (Xen35) containing the stkP allelic variant. Amino acids in red represent the amino acids deleted in the StkP allelic variant. B- shows the StkP amino acid sequence in Xen 35, with the 3rd PASTA domain not present, and in dark blue the remaining 10 amino acid repeat. (TIF) [file pone.0127212.s001.tif]

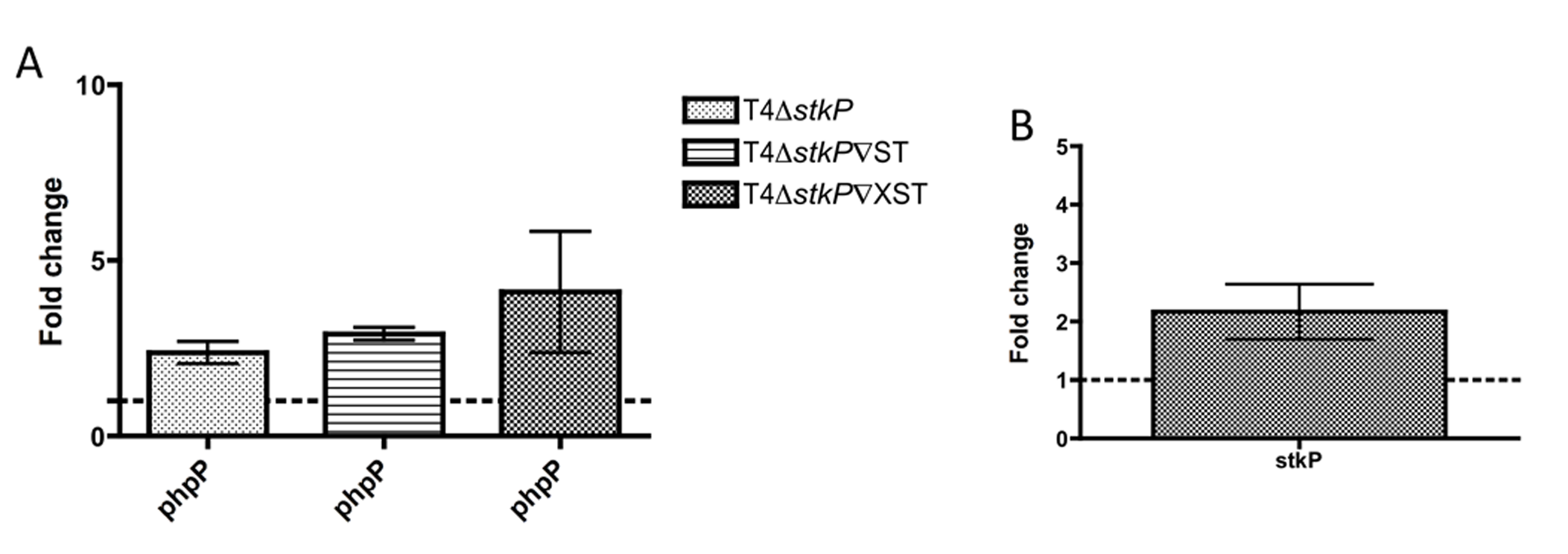

Supplement: S2 Fig — A- Graph shows RT-PCR expression of phpP in T4ΔstkP, T4ΔstkP∇ST and T4ΔstkP∇XST compared to TIGR4. Fold change represents that of the mutant strain compared to TIGR4. Each bar represents the average of three replicas and errors bars the standard deviation. B- Shows RT-PCR expression of stkP in T4ΔstkP∇XST compared to T4ΔstkP∇ST. Fold change represents that of T4ΔstkP∇XST compared to T4ΔstkP∇ST. Each bar represents the average of three replicas and errors bars the standard deviation. (TIF) [file pone.0127212.s002.tif]

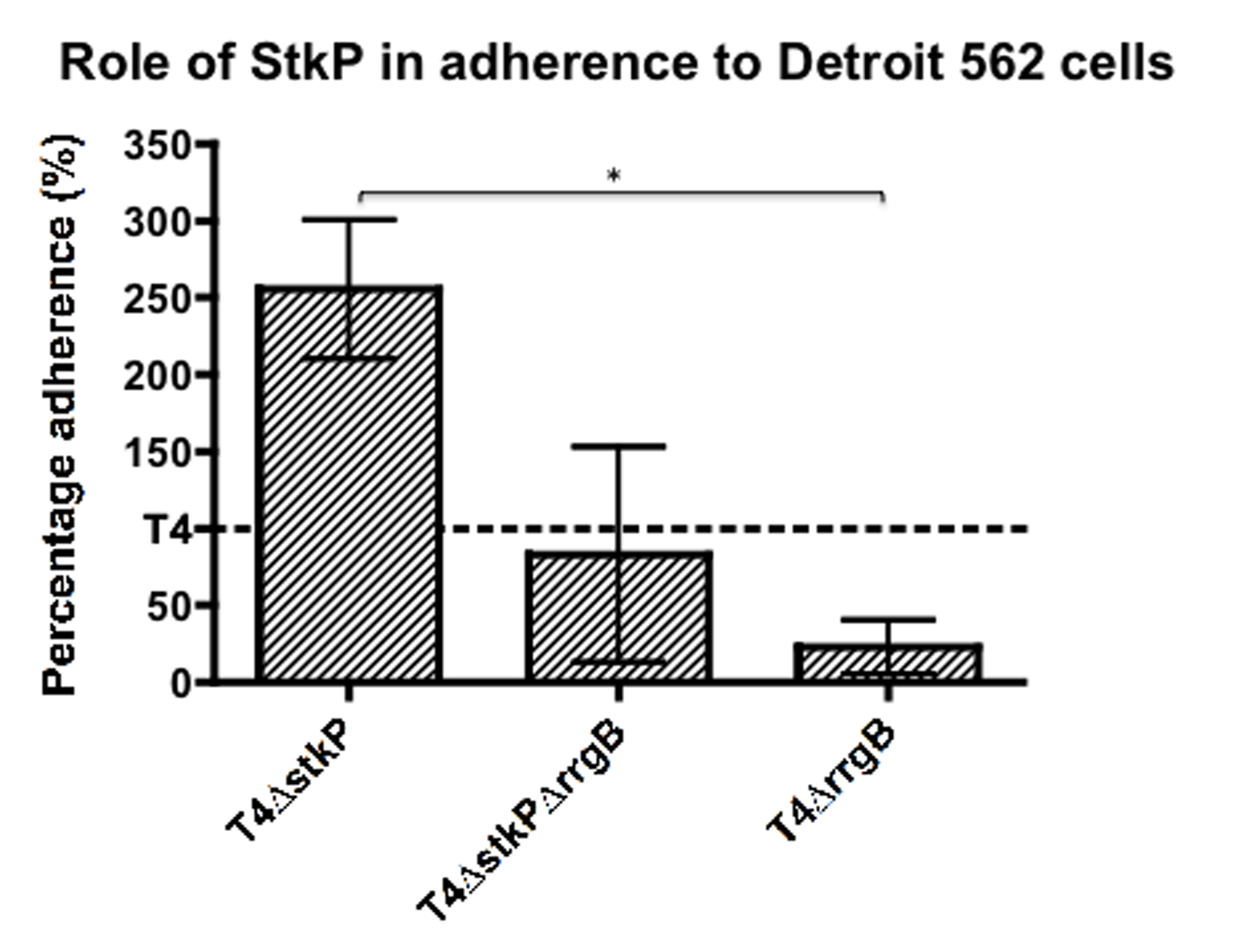

Supplement: S3 Fig — Adherence of strains TIGR4, T4ΔstkP, T4ΔstkPΔrrgB and T4ΔrrgB was assessed to Detroit 562 cell lines. Data is represented as percentage adherence relative to that of TIGR4 (100%, dashed line). Each bar is an average of at least two replicates and the error bars represent the standard error of the mean. Statistical analysis was performed using a 1-way ANOVA with a Tukeys testing correction, * P<0.01. (TIF) [file pone.0127212.s003.tif]
