# Supplementary material for: A Serine-Threonine Kinase (StkP) Regulates Expression of the Pneumococcal Pilus and Modulates Bacterial Adherence to Human Epithelial and Endothelial Cells In Vitro
Source: PLoS One. 2015 Jun 19;10(6):e0127212. doi: 10.1371/journal.pone.0127212 (PMC4474723; doi:10.1371/journal.pone.0127212)
Supplement: S1 Table — (PDF) [file pone.0127212.s004.pdf]

Table S1. Strains and plasmids used in this study

Strain name	Description	Reference
TIGR4	Serotype 4, TIGR4 strain	[1,2]
Xen35	Serotype 4, TIGR4 derivative	[3]
T4Δ*stkP*	s*tkP* deletion mutant in TIGR4, contains transpson insertion with a spectinomycin resistance cassette.	This study
T4Δ*rrgB*	*rrgB* deletion in TIGR4, contains full removal of *rrgB* gene replaced with a kanamycin resistance cassette.	This study
T4Δ*stkP*Δ*rrgB*	T4Δ*stkP* with *rrgB* gene replaced with a kanamycin resistance cassette.	This study
T4Δ*stkP*∇ST (also referred to as T4Δ*stkP* ST)	T4Δ*stkP* with full length StkP inserted into SP_1886 under the control of a strong promoter. Constructed using plasmid pCP2 ST.	This study
T4Δ*stkP*∇XST (also referred to as T4Δ*stkP* XST)	T4Δ*stkP* with the PASTA domain 3 deletion StkP inserted into SP_1886 under the control of a strong promoter. Constructed using plasmid pCP2 XST.	This study
pCEP2	Chromosomal expression platform	[4]
pC2LSD P2	Intermediate in construction of pCP2 ST and pCP2 XST.	This study

pCP2 ST	Plasmid containing T4 StkP downstream of a strong promoter (promoter of SP_2012), chloramphenicol resistance cassette.	This study
pCP2 XST	Plasmid containing allelic variant StkP downstream of a strong promoter (promoter of SP_2012), chloramphenicol resistance cassette.	This study
pR412	Used for i*n vitro* mariner mutagenesis, contains a spectinomycin resistance cassette flanked by inverted repeats.	[5]

1. Aaberge IS, Engz J, Lermark G, Levik M (1995) Virulence of Streptococcuspneumoniaein mice: a standardized method for preparation and frozen storage of the experimental bacterial inoculum. Microbial Pathogenesis: 141-152.
2. Tettelin H, Nelson KE, Paulsen IT, Eisen JA, Read TD, et al. (2001) Complete genome sequence of a virulent isolate of Streptococcus pneumoniae. Science 293: 498-506.
3. Orihuela CJ, Gao G, McGee M, Yu J, Francis KP, et al. (2003) Organ-specific models of Streptococcus pneumoniae Disease. Scandinavian Journal of Infectious Diseases 35: 647-652.
4. Guiral S, Henard V, Laaberki MH, Granadel C, Prudhomme M, et al. (2006) Construction and evaluation of a chromosomal expression platform (CEP) for ectopic, maltose-driven gene expression in Streptococcus pneumoniae. Microbiology 152: 343-349.
5. Lampe DJ, Akerley BJ, Rubin EJ, Mekalanos JJ, Robertson HM (1999) Hyperactive transposase mutants of the Himar1 mariner transposon. Proc Natl Acad Sci USA 96: 11428-11433.
