# Supplementary material for: A Serine-Threonine Kinase (StkP) Regulates Expression of the Pneumococcal Pilus and Modulates Bacterial Adherence to Human Epithelial and Endothelial Cells In Vitro
Source: PLoS One. 2015 Jun 19;10(6):e0127212. doi: 10.1371/journal.pone.0127212 (PMC4474723; doi:10.1371/journal.pone.0127212)
Supplement: S2 Table — (PDF) [file pone.0127212.s005.pdf]

Table S2. Primers and related information used for construction of knockout strains via in vitro mariner mutagenesis and splice overlap PCR and also primers used to construct plasmids pCP2 ST and pCP2 XST.

Gene	Gene number	Primer name	DNA sequence 5'-3'	Size (bp)
stkP	SP_1732	StkPF FL	ATGATCCAAATCGGCAAGA	1980
		StkPR	TTAAGGAGTAGCTGAAGTTG	
stkP	SP_1732	STKP COMP F*	CACTACCATGGATGATCCAAATCGGCAAG	1980 (T4)
		STKP COMP R	TTTCAGGGATCCTTAAGGAGTAGCTGAAGTTG	1764 (variant)
Chlor cassette		CHLOR ST C F	CCTTAAGGATCCCTGAAAAATTTGTTTGATTT	850
		CHLOR COMP R*	CTTCCCCGATATCTTATAAAAGCCAGTCATTAG	
INT SP_2012-SP_2013	n/a	P2 F	CTAGGCCTTAAGGGATTCCTTGGTTTAC	176
	n/a	P2 R	TAGCCATGGTAGTGATTTCCTCCTTATG	
rrgA	SP_0462	0462F-25C*	CAAGATGGTACTATAACG	1350
		0462 R	TCCCCAGCTTGGATTTCTCCTTATTCATATC	
Kan cassette		KANGB-F	AGGAGAAATCCAAGCTGGGGATCCGTTTG	795
		KANGB-R	CCTTTCTCTCTTACCTAAAACAATTCATCCAGTAA	

rrgC	SP_0464	0464F	ATGAATTGTTTTAGGTAAGAGAGAAAGGAGCCATT	807
		0464 R-34R*	TTCTGATTGACAACCGTAATCG	
		MP127	CCGGGGACTTATCAGCCAACC	
		MP128	TACTAGCGACGCCATCTATGTG	
amiF	SP_1886	amiFF T4G	AGGATGAAGAAGAACGTAAAG	
