# Supplementary material for: A Serine-Threonine Kinase (StkP) Regulates Expression of the Pneumococcal Pilus and Modulates Bacterial Adherence to Human Epithelial and Endothelial Cells In Vitro
Source: PLoS One. 2015 Jun 19;10(6):e0127212. doi: 10.1371/journal.pone.0127212 (PMC4474723; doi:10.1371/journal.pone.0127212)
Supplement: S3 Table — (PDF) [file pone.0127212.s006.pdf]

Table S3. Primers and related information used for RT-PCR analysis of pilus, *stkP* and *phpP* expression in T4Δ*stkP,* T4Δ*stkP*∇ST and T4Δ*stkP*∇XST*.*

Gene	Gene number	Primer name	DNA sequence 5'-3'	Size (bp)
rlrA	SP_0461	0461 RT-F	CCATCGCAACAGGCTACC	185
		0461 RT-R	TGTGACCCAATCCATACTTCC	
rrgA	SP_0462	0462 RT-F	AACCAGTCCAGCGATAGG	185
		0462 RT-R	CTTCTGTCAAGGTGTATGTCC	
rrgB	SP_0463	0463 RT-F	ATACACCTGTGAACCACCAAG	104
		0463 RT-R	CATTCTATCGCTCCAGTTTGC	
rrgC	SP_0464	0464 RT-F	GTATCTTCTTTGTTATGGCTCTG	185
		0464 RT-R	ATCATCATAGGAATACGAATCATC	
srtB	SP_0466	0466 RT-F	GGTGTCTCGCTTGTATTATCG	86
		0466 RT-R	TGTCAGCCTCATCCAACG	
srtC	SP_0467	0467 RT-F	GTGTCTCGTTATTATTATCGTATTG	91
		0467 RT-R	CCTCAAGTTCTGCCTTATCC	
srtD	SP_0468	0468 RT-F	TCTCGCCTACAATCAACGC	169
		0468 RT-R	ATAATCTGCTCCCAAATAAACCG	
gyrA	SP_1219	gyrA RT-F	GCGCGAGCTCTTCCTGATGT	100
		gyrA RT-R	TATGGGGTTTGTCTGGGGTC	
phpP	SP_1733	*phpP* RT F	GATGTTGGTCAGAAACGAACAA	702
		phpP RT R	GTTCATAGAAACAAGGGCAACC	
stkP	SP_1732	StkP RT-F	AAACAGATTGGTCGAGGAGGTA	758
		StkP RT R	TCCACATACATCTCTGAAACCG	
